# Development of CVD Silica Membranes Having High Hydrogen Permeance and Steam Durability and a Membrane Reactor for a Water Gas Shift Reaction

**DOI:** 10.3390/membranes9110140

**Published:** 2019-10-30

**Authors:** Ryoichi Nishida, Toshiki Tago, Takashi Saitoh, Masahiro Seshimo, Shin-ichi Nakao

**Affiliations:** Inorganic Membranes Research Center, Research Institute of Innovative Technology for the Earth (RITE), Kyoto 619-0237, Japan

**Keywords:** hydrogen, water gas shift reaction, amorphous silica membrane, counter-diffusion CVD, membrane reactor

## Abstract

Water gas shift reaction of carbon monoxide (CO) with membrane reactors should be a promising method for hydrogen mass-production because of its high CO conversion, high hydrogen purity and low carbon dioxide emission. For developing such membrane reactors, we need hydrogen permselective membranes with high hydrogen permeance with order of 10^−6^ mol m^−2^ s^−1^ Pa^−1^ at 573 K and high steam durability. In this study, we have optimized the kind of substrates, precursors, vapor concentration, and chemical vapor deposition (CVD) time using the counter-diffusion CVD method for developing such membranes. The developed membrane prepared from hexamethyldisiloxane has a hydrogen permeance of 1.29 × 10^−6^ mol m^−2^ s^−1^ Pa^−1^ at 573 K and high steam durability. We also conducted water gas shift reactions with membrane reactors installed the developed silica membranes. The results indicated that reactions proceed efficiently with the conversion around 95–97%, hydrogen purity around 94%, and hydrogen recovery around 60% at space velocity (SV) 7000.

## 1. Introduction

Hydrogen energy is very attractive from the viewpoint of global warming prevention. Since hydrogen is a secondary energy, its production method is important. For no emission of carbon dioxide, the most preferable method is the electrolysis of water using renewable energy such as solar power and wind power. However, in many cases, the location of hydrogen production by renewable energy does not coincide with the location of hydrogen energy utilization. Therefore, at present, the most common hydrogen production method is steam reforming of methane followed by water gas shift reaction of carbon monoxide. Although this method has already been industrially established, there is a problem that it is not carbon dioxide free. Since the steam reforming reaction of methane is an endothermic reaction, the heat of the reaction needs to be supplied, which is accompanied by the emission of carbon dioxide. Since the water gas shift reaction of carbon monoxide is an exothermic reaction, carbon dioxide emissions are very much reduced. As a large amount of carbon monoxide source, there are coke furnace gas and blast furnace gas (BFG), and coal gasification gas also becomes a carbon monoxide source.

In hydrogen production by these reactions, the product gases are hydrogen and carbon dioxide, and small amounts of unreacted methane and carbon monoxide. Therefore, separation and purification steps are required to use hydrogen, and a pressure swing adsorption method is usually employed to produce high purity hydrogen gas. On the other hand, in recent years, membrane reactors capable of simultaneously performing reaction and separation have attracted attention [1,2,3,4,5,6]. When a membrane reactor is used, the apparatus becomes compact, and further, it is possible to shift the thermodynamic reaction equilibrium to the production side by selectively permeating the desired product components. As the result reaction conversion is improved. The development issue is the development of membranes with excellent permselectivity of the target component.

**Figure 1:**
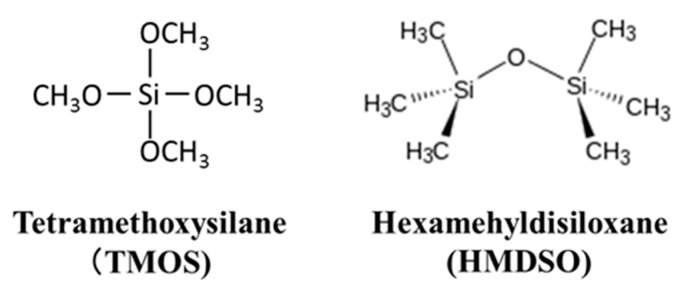
Molecular structures of tetramethoxysilane and hexamethyldisiloxane.

For hydrogen separation, palladium membrane or silica membrane having selective permeability to hydrogen is used [7,8,9,10,11], but since the water gas shift reaction temperature is usually 523 K to 573 K, hydrogen permeability of a conventional palladium membrane or silica membrane is low and it is difficult to use in practical processes. The silica membranes are prepared by a sol–gel method [12,13] or a chemical vapor deposition (CVD) method [14,15,16], but the membrane prepared by the CVD method is superior from the viewpoint of water vapor resistance [17,18]. A CVD membrane having separation properties for hydrogen and carbon dioxide has been developed by using two types of precursors, tetra-methoxysilane (TMOS) or hexamethyldisiloxane (HMDSO) as shown in Figure 1, conventionally a hydrogen permeance about 1 × 10^−7^ mol m^−2^ s^−1^ Pa^−1^ at 773 to 873 K, and a H_2_/N_2_ selectivity of 1000 to 2000 [19], which is almost the same as H_2_/CO_2_ selectivity. With the selectivity of 1000 to 2000, the obtained purity of permeate hydrogen is more than 99.9%. The membranes having high selectivity usually have low permeance and on the other hand the membranes having low selectivity show high permeance. In several applications of hydrogen, high purity such as more than 99.9% is not required and a purity of about 90%–95% is enough. When the membrane is applied to the water gas shift reaction, the membrane must have steam durability. It was reported that the membranes derived from TMOS and HMDSO have the durability, but it is not high enough.

In this study, CVD silica membranes having both high hydrogen permeance with an order of 10^−6^ mol m^−2^ s^−1^ Pa^−1^ at 573 K and selectivity, which is enough to obtain hydrogen purity of 90–95% and high steam durability were developed. Then the water gas shift reaction with a membrane reactor installed the developed CVD silica membrane was investigated.

## 2. Materials and Methods

### 2.1. Preparation and Permeation Measurements of Silica Membranes Derived from TMOS or HMDSO

**Figure 2:**
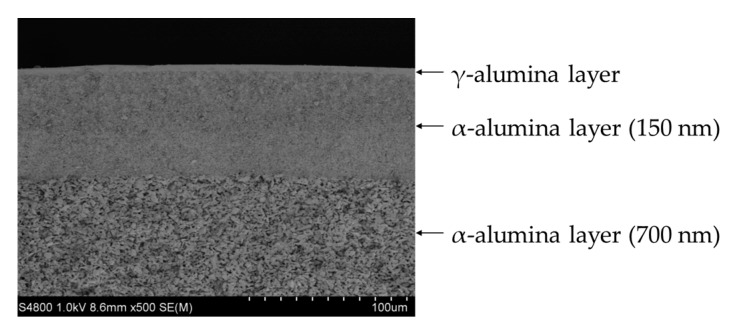
The SEM image of γ-alumina layer on the asymmetric substrate.

**Figure 3:**
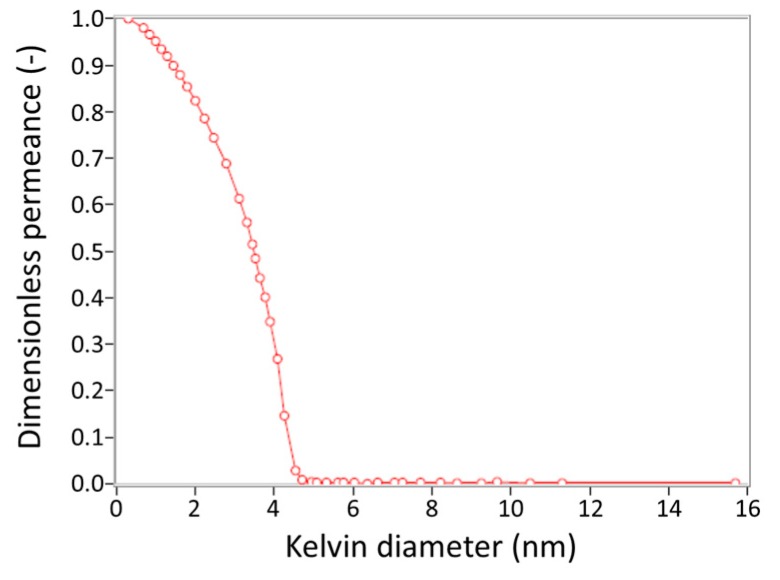
The nanopermporometry measurements of γ-alumina layer on the asymmetric substrate.

Two kinds of porous α-alumina substrate tube (diameter of 6 mm, length of 100 mm.) were purchased from Noritake Co. (Nagoya, Japan), one is a symmetric tube with 150 nm pores (hydrogen permeance; 5.12 × 10^−6^ mol m^−2^ s^−1^ Pa^−1^, nitrogen permeance.: 1.67 × 10^−6^ mol m^−2^ s^−1^ Pa^−1^ at 303 K) and another is an asymmetric one with 150 nm/700 nm pores (hydrogen permeance; 3.50 × 10^−5^ mol m^−2^ s^−1^ Pa^−1^, nitrogen permeance: 1.20 × 10^−5^ mol m^−2^ s^−1^ Pa^−1^ at 303 K). A membrane was prepared at the center of substrate (70 mm), and the other parts were glazed with sealant. A γ-alumina layer was applied to the substrate surface to reduce the pore size, according to the report by Yoshino et al. [20]. The outer surface of the effective area of the substrate was dipped in a boehmite sol (γ-AlOOH) for 5 seconds and then dried for 1 h in the air and calcined at 873 K for 3 h. The coating process was repeated three times. The γ-alumina layer was uniformly coated on the substrate as shown in Figure 2 and its pore size was determined to be around 4 nm by nanopermporometry measurements as shown in Figure 3.

**Figure 4:**
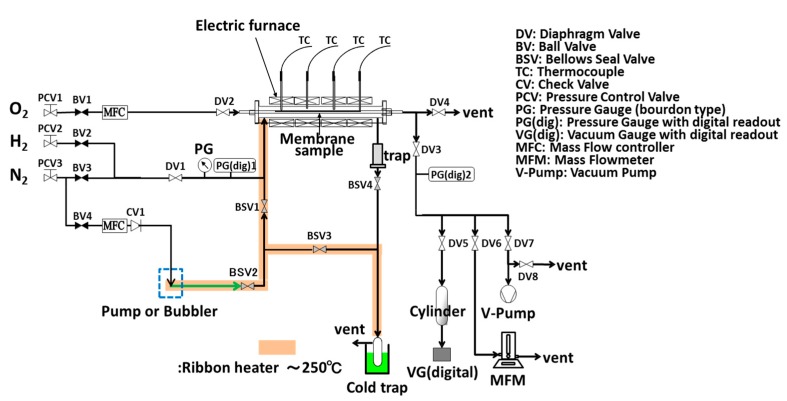
A schematic diagram of chemical vapor deposition (CVD) and the permeation evaluation apparatus.

An amorphous silica layer was deposited on the γ-alumina layer by counter-diffusion chemical vapor deposition (CVD). A schematic diagram of the experimental apparatus for preparing TMOS or HMDSO-derived silica membranes and evaluating their permeation performance is shown in Figure 4.

TMOS or HMDSO (Shin-Etsu Chemical Co. Ltd., Tokyo, Japan) vapor was supplied by a bubbler or a syringe pump (KDS-410, KD Scientific, Tokyo, Japan) in a nitrogen (200 mL min^−1^) carrier gas, from the outside of the γ-alumina-coated membrane substrate, and O_2_ (20 mL min^−1^) was supplied from the inside and CVD was conducted at the reaction temperature of 873 K.

To examine the influence of silica precursors, silica membranes were deposited to γ-alumina coated symmetric substrates with the precursor concentration around 0.85 mol m^−3^ (0.886 mol m^−3^ for HMDSO and 0.804 mol m^−3^ for TMOS) for 5 min as CVD time.

An asymmetric substrate coated with γ-alumina was deposited with 0.885 mol m^−3^ HMDSO to examine the effect of substrate.

Regarding the influence of precursor concentration and CVD time of HMDSO, first, CVD was conducted in which the precursor concentration was lowered from 0.885 mol m^−3^ to 0.584 mol m^−3^. Next, the CVD time was shortened from 5 min to 3 min with a precursor concentration of 0.624 mol m^−3^.

Membrane gas permeation performance was evaluated at 773, 673, and 573 K using single-component gases of hydrogen and nitrogen. Their permeation was measured with a bubble flow meter (SF 1U, Horiba Co., Kyoto, Japan).

### 2.2. Steam Durability Evaluation of Silica Membranes Derived from HMDSO

**Figure 5:**
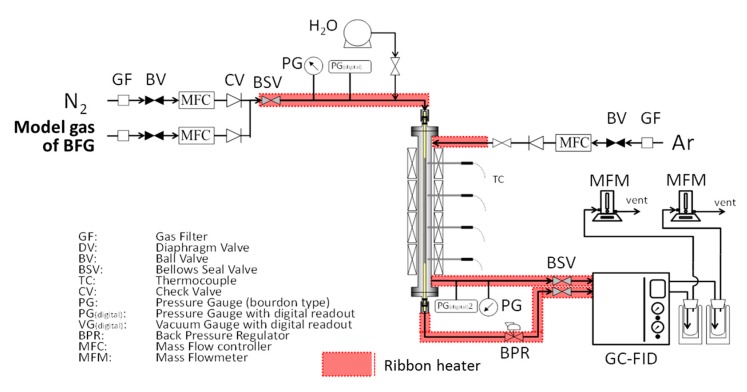
A schematic diagram of membrane reactor for the water gas shift reaction.

Steam durability evaluation of silica membranes derived from HMDSO was conducted with using the apparatus for the water gas shift reaction as shown in Figure 5.

The evaluated HMDSO-derived silica membrane was prepared with precursor vapor concentration of 0.73 mol m^-3^ and CVD time of 5 min. Its hydrogen permeance was 1.1 × 10^−6^ mol m^−2^ s^−1^ Pa^−1^ and selectivity of H_2_/N_2_ = 159 at 573 K at just after preparation. A mixture of nitrogen and water was supplied into the inside of the silica membrane tube, and nitrogen gas was carried outside the silica membrane. Gas flow rates of nitrogen inside, water, and nitrogen for sweep were 54, 20, and 50 mL min^−1^ respectively. The feed gas pressure was 305 kPa, and the permeate pressure was 100 kPa and temperature was fixed at 573 K.

Membrane gas permeation performance was evaluated at 773, 673, and 573 K using single-component gases of hydrogen, nitrogen by a bubble flow meter (SF 1U, Horiba Co., Kyoto, Japan).

### 2.3. Water Gas Shift Reaction with Membrane Reactors Installed Developed Silica Membranes

Figure 5 shows a schematic diagram of a membrane reactor for the water gas shift reaction. Employed HMDSO-derived silica membrane had a hydrogen permeance of 0.98 × 10^−6^ mol m^−2^s^−1^ Pa^−1^ and selectivity of H_2_/N_2_ = 212 at 573 K. Cu/ZnO/Al_2_O_3_ granules (C18-7, Sud Chemie Catalysts, Munich, Germany) were employed as the catalyst. A catalyst (1.05 g) was put into the HMDSO-derived silica membrane tube, and model gas of BFG, which contained 52% of N_2_, 22% of CO, 22% of CO_2_, and 4% of H_2_ was fed into the tube.

The reaction was conducted at the temperature of 573 K, and the feed and permeate pressure were 305 kPa and 100 kPa respectively. On the permeation side of the membrane, 49.4 mL min^−1^ of argon as a sweep gas was flowed. The CO conversion was determined from the concentration of unreacted CO at the outlet of the feed side. The hydrogen recovery rate was calculated using the ratio of the amount of hydrogen at the outlet, based on the sum of hydrogen and carbon monoxide at the inlet as hydrogen. Hydrogen purity was calculated by the ratio of detected concentration by gas chromatography (7820A, Agilent Technologies, Santa Clara, CA, USA) excluding argon and hydrogen.

TMOS-derived silica membrane was also employed for the water gas shift reaction in the same manner.

## 3. Results and Discussion

### 3.1. Permeance of Silica Membranes Derived from TMOS or HMDSO

**Figure 6:**
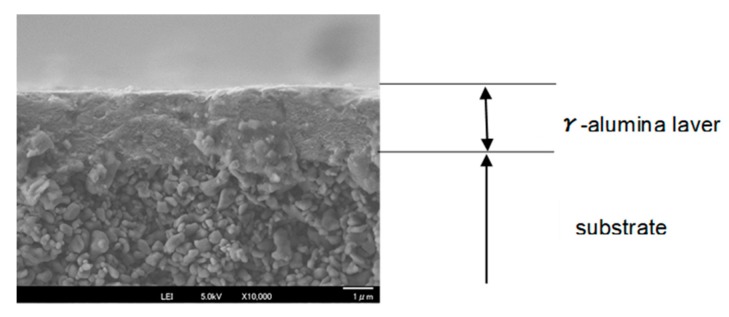
The SEM image of silica membrane deposited in pores of the γ-alumina layer on the substrate.

Figure 6 shows the SEM image of silica membrane deposited in pores of the γ-alumina layer on the substrate by counter-diffusion CVD.

**Figure 7:**
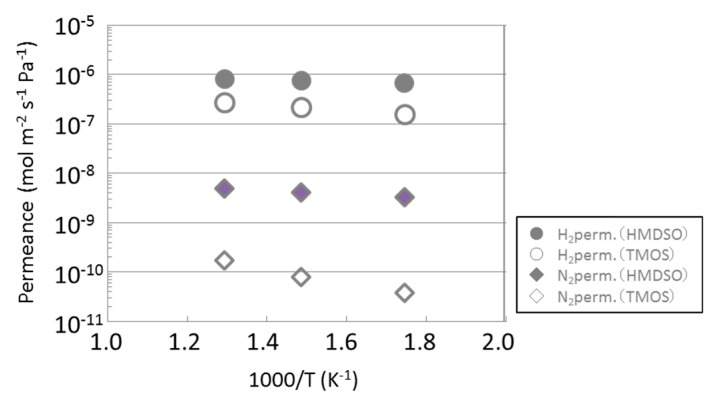
An Arrhenius plot of hydrogen and nitrogen permeances through HMDSO-derived membrane and TMOS-derived membrane. Membranes were deposited on symmetric substrates for 5 min as CVD time with precursor concentration around 0.85 mol m^−3^. The selectivity (H_2_/N_2_) was 209 for HMDSO-derived silica membrane and 4230 for TMOS-derived one.

Figure 7 is an Arrhenius plots of single gas permeance though both membranes. The single-gas permeance of HMDSO-derived silica membrane was bigger than that of TMOS-derived one and activated energy of the HMDSO-derived membrane was smaller than that of the TMOS-derived one. These results indicate that the pore size of the HMDSO-derived membrane was larger than that of the TMOS-derived one, and it should be closed to the diameter of nitrogen molecule. This pore size increased nitrogen permeance significantly comparing to hydrogen permeance. Therefore the selectivity (H_2_/N_2_) of the HMDSO-derived membrane became lower than that of TMOS-derived one.

**Figure 8:**
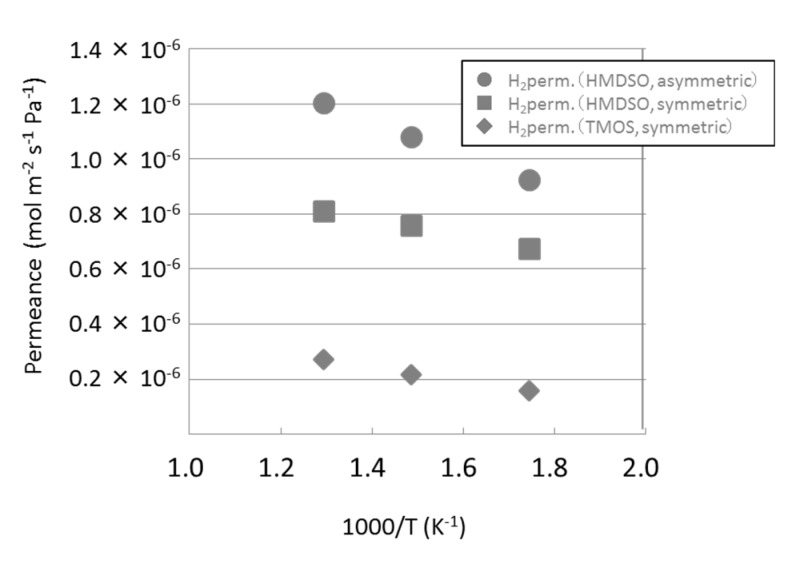
Influence of the substrate to hydrogen permeance of silica membranes. The membranes were deposited for 5 min as CVD time with a precursor concentration of about 0.885 mol m^−3^.

As shown in Figure 7, hydrogen permeance was less than 1 × 10^−6^ mol m^−2^ s^−1^ Pa^−1^. Therefore we examined two types of substrate tubes for HMDSO-derived membrane, one was a symmetrical type with 150 nm pores and the other was an asymmetric type with 150 nm pores as the upper layer and 700 nm pores as the lower layer. The asymmetric one had lower permeation resistance comparing to symmetric one as described in the Section 2.1. The results are shown in Figure 8 and Table 1. For reference, TMOS with a symmetrical substrate was also plotted. As a result, hydrogen permeance with an asymmetric substrate was 9.25 × 10 ^−7^ mol m^−2^ s^−1^ Pa^−1^, that was about 1.4 times of symmetrical type substrate. It was confirmed that the asymmetric substrate increased the hydrogen permeance of silica membranes by its lower permeation resistance.

**Figure 9:**
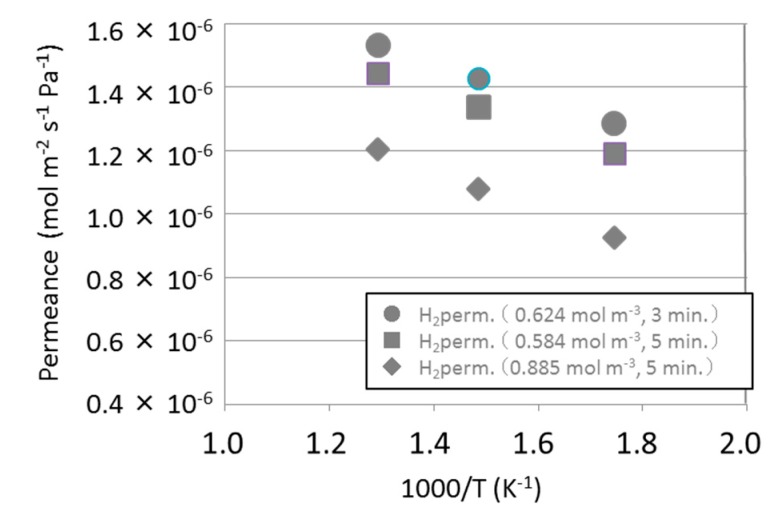
Influence of precursor concentration and CVD time to hydrogen permeance through HMDSO-derived membranes deposited on asymmetric substrates.

The hydrogen permeance at 573 K was still less than 1 × 10^−6^ mol m^−2^ s^−1^ Pa^−1^ as shown in Figure 9 and Table 2. Then concentration of precursor and time of CVD to prepare membranes were changed to obtain high hydrogen permeance. As shown in Figure 9, reducing the precursor vapor concentration and shortening the CVD time improved hydrogen permeance by about 1.4 times and the permeance of 1.29 × 10^−6^ mol m^−2^ s^−1^ Pa^−1^ at 573 K was obtained.

As shown in Table 3, the newly developed HMDSO-derived silica membrane had much higher hydrogen permeance than other silica membranes which were expected to have steam durability [19,21,22,23,24]. The selectivity (H_2_/N_2_) was relatively small, however, they were enough for several applications.

The permeation resistance of asymmetric substrate was smaller than symmetric one. The result indicates that the permeation resistance of substrates had great influence on the permeation performance of silica membranes, and we could get higher hydrogen permeance with the asymmetric substrate. In addition, the precursor concentration and CVD time had also influence on the hydrogen permeance. Since the higher hydrogen permeance was obtained with lower precursor concentration and shorter CVD time, the thickness of silica membrane was supposed to be thinner. However, further experiments and analysis were required for a clear conclusion.

### 3.2. Steam Durability of Silica Membranes Derived from HMDSO

**Figure 10:**
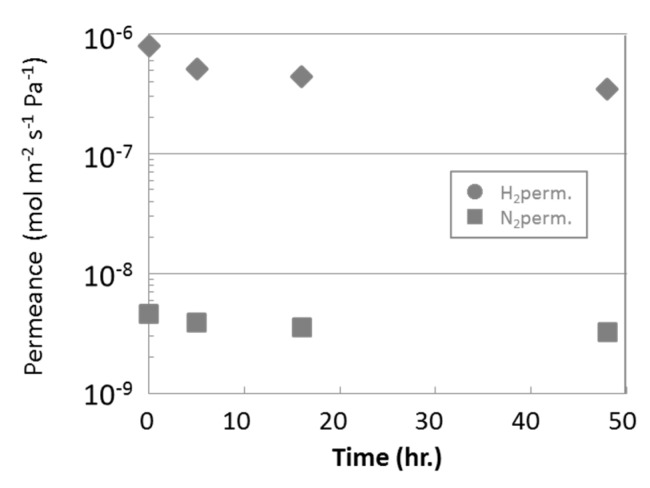
Steam durability evaluation of HMDSO-derived membrane.

Figure 10 shows steam durability of the HMDSO-derived silica membrane. The hydrogen permeance was lowered in the initial several hours, however, there was almost no decline after that. Just after membrane preparation, a lot of silanol groups remained in the deposited silica membrane. As the condensation reaction of silanol groups progressed, the silica membrane became denser to give lower hydrogen permeance. During the same time, the amount of silanol groups decreased, so that densification did not occur and the hydrogen permeance became constant.

Therefore, it was succeeded to develop a HMDSO-derived silica membrane having both high hydrogen permeance more than 1 × 10^−6^ mol m^−2^ s^−1^ Pa^−1^ and good steam durability.

### 3.3. Water Gas Shift Reaction with Membrane Reactors

**Figure 11:**
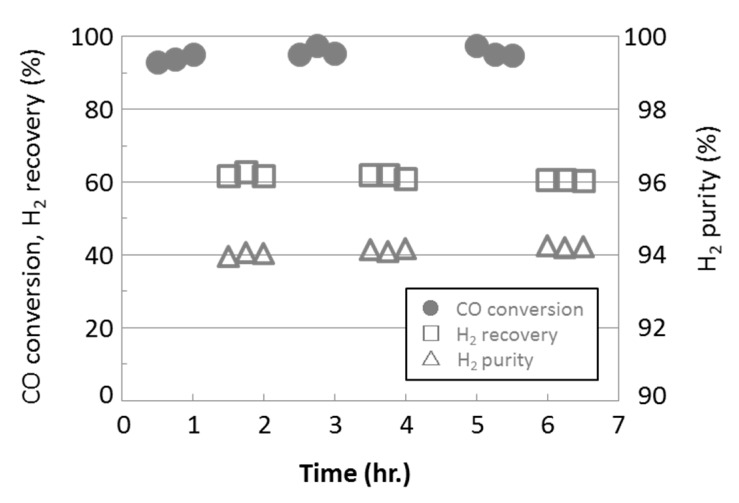
Water gas shift reaction by membrane reactor installed the developed HMDSO-derived silica membrane.

The results of a water gas shift reaction with a membrane reactor installed the developed HMDSO-derived silica membrane are shown in Figure 11.

When the reaction was operated at space velocity (SV) = 7000 for 7 h, CO conversion, hydrogen recovery, and hydrogen purity were about 95 to 97%, about 60%, and around 94% respectively.

**Figure 12:**
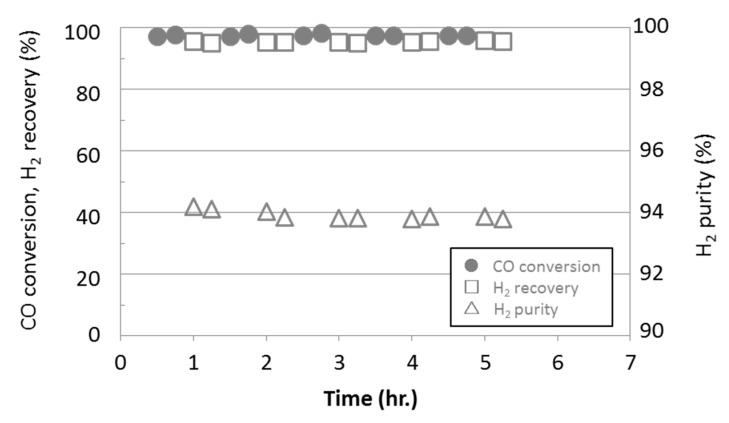
Water gas shift reaction by membrane reactor installed the TMOS-derived silica membrane.

For comparison, the water gas shift reaction with a membrane reactor installed the TMOS-derived silica membrane were also conducted and the results are shown in Figure 12.

The hydrogen recovery with HMDSO-derived silica membrane was 1.5 times higher than that with TMOS-derived one even though hydrogen purity was lower. The higher hydrogen permeance of the developed HMDSO-derived silica membrane should have given this higher hydrogen recovery.

**Figure 13:**
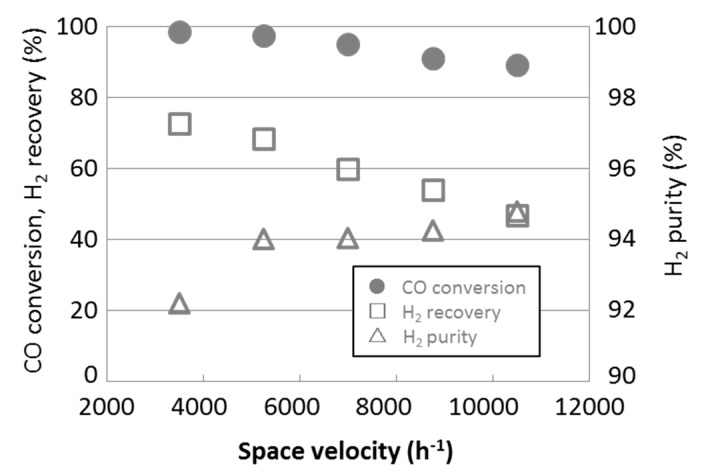
Water gas shift reaction with various space velocities (SVs).

The influence of the SV was evaluated by changing from 3500 to 10,500, and the result was summarized in Figure 13.

As shown in the Figure 13, hydrogen recovery rate decreased constantly as SV increased and it became less than 50% at SV = 10,500. We also see that the hydrogen purity was improved at SV = 10,500. This was probably due to a mismatch between the permeance of the developed silica membrane and high SV condition. For the developed membrane, SV such as 10,500 seems to be too high to permeate hydrogen efficiently. Much of the amount of hydrogen might remain at the retentate side even at the end of membrane tube, so the hydrogen partial pressure along the longitudinal direction of the membrane tube at SV = 10,500 decreased more gradually than the pressure at lower SV. The still high hydrogen partial pressure at the outlet of the membrane might prevent impurity to permeate through the membrane, so hydrogen purity was higher at the high SV such as 10,500.

Optimization of operating conditions might be required in consideration of CO conversion rate, hydrogen recovery rate, and hydrogen purity according to the application.

## 4. Conclusions

We succeeded in developing a silica membrane having both high hydrogen permeance, 1.29 × 10^−6^ mol m^−2^ s^−1^ Pa^−1^, and high steam durability. When we applied the membrane reactor installed the developed membrane to the water gas shift reaction of the BFG model gas, the reaction was conducted efficiently with CO conversion of 95–97%, hydrogen recovery of about 60%, and hydrogen purity about 94% at space velocity (SV) = 7000. We also evaluated the influence of SV changing from 3500 to 10,500 to the reaction. The results showed that CO conversion and hydrogen recovery rate became lower from 99 to 89% and from 73 to 47%, respectively, and hydrogen purity became higher from 92 to 95% as the SV increased.

## Figures and Tables

**Table 1:** Influence of the substrate to the selectivity (H_2_/N_2_) of silica membranes. The membranes were deposited for 5 min as CVD time with a precursor concentration of about 0.885 mol m^−3^.

Samples	Selectivity (H_2_/N_2_)
HMDSO-derived silica membrane (asymmetric substrate)	265
HMDSO-derived silica membrane (symmetric substrate)	209
TMOS-derived silica membrane (symmetric substrate)	4230

**Table 2:** Influence of precursor concentration and CVD time to the selectivity (H_2_/N_2_) of the HMDSO-derived membranes deposited on asymmetric substrates.

Samples	Selectivity (H_2_/N_2_)
HMDSO-derived silica membrane (0.624 mol m^−3^, 3 min)	96
HMDSO-derived silica membrane (0.584 mol m^−3^, 5 min)	107
HMDSO-derived silica membrane (0.885 mol m^−3^, 5 min)	265

**Table 3:** The comparison table of hydrogen permeances and selectivity values of silica membranes, which are expected to have steam durability (at 573 K).

Samples	H_2_ Permeance (mol m^−2^ s^−1^ Pa^−1^)	Selectivity (H_2_/N_2_)
HMDSO-derived silica membrane (This research)	1.29 × 10^−6^	96
TMOS-derived silica membrane (This research)	1.6 × 10^−7^	4230
HMDSO-derived silica membrane [19]	2.0 × 10^−7^	4600
TMOS-derived silica membrane [21]	1.0 × 10^−7^	>2000
Cobalt oxide silica membrane [22]	1.9 × 10^−7^	1500 (H_2_/CO_2_) **
Magnesium-diped silica membrane [23]	7 × 10^−8^	350 (H_2_/CO_2_) **
BTESE*-derived organosilica membrane [24]	4.6 × 10^−8^	18 (H_2_/CO_2_) **

* BTESE; 1,2-bis(toriethoxysilyl)ethane; ** Selectivity (H_2_/N_2_) is as almost same as selectivity (H_2_/CO_2_).

## References

[1] Sharma R., Kumar A., Upadhyay R.K. (2017). Performance comparison of methanol steam reforming integrated to Pd-Ag membrane: Membrane reformer vs. membrane separator. Sep. Purif. Technol..

[2] Ma L.C., Castro-Dominguez B., Kzantzis N.K., Ma Y.H. (2016). A cost assessment study for a large-scale water gas shift catalytic membrane reactor module in the presence of uncertainty. Sep. Purif. Technol..

[3] Amanipour M., Towfighi J., Babakhani E.G., Heidari M. (2016). H_2_ permeation properties of a catalytic membrane reactor in methane steam reforming. Int. J. Hydrog. Energy.

[4] Akamatsu K., Tago T., Seshimo M., Nakao S.I. (2015). Long-term stable H_2_ production from methylcyclohexane using a membrane reactor with dimethoxydiphenylsilane-derived silica membrane prepared via chemical vapor deposition. Ind. Eng. Chem. Res..

[5] Lima F.V., Daoutidis P., Tsapatsis M. (2016). Modeling, optimization, and cost analysis of an IGCC plant with a membrane reactor for carbon capture. AIChE J..

[6] Dong X., Wang H., Rui Z., Lin Y.S. (2015). Tubular dual-layer MFI zeolite membrane reactor for hydrogen production via the WGS reaction: Experimental and modeling studies. Chem. Eng. J..

[7] Allemand M., Martin M.H., Reyter D., Roue L., Guay D., Botton G. (2011). Synthesis of Cu-Pd alloy thin films by co-electrodeposition. Electrochem. Acta.

[8] Seshimo M., Ozawa M., Sone M., Sakurai M., Kameyama H. (2008). Fabrication of a novel Pd/γ-alumina graded membrane by electroless plating on nanoporous γ-alumina. J. Membr. Sci..

[9] Diniz da Costa J.C., Lu G.Q., Rudolph V., Lin Y.S. (2002). Novel molecular sieve silica (MMS) membranes: Characterization and permeation of single-step and two-step sol-gel membranes. J. Membr. Sci..

[10] Kanezashi M., Shioda T., Gunji T., Tsuru T. (2012). Gas permeation properties of silica membranes with uniform pore size derived from polyhedral oligomeric silsesquioxane. AIChE J..

[11] Seshimo M., Akamatsu K., Furuta S., Nakao S.-I. (2015). Comparative study of the influence of toluene and methylcyclohexane on the performance of dimethoxydiphenylsilane-derived silica membranes prepared by chemical vapor deposition. Sep. Purif. Technol..

[12] Cao G., Lu Y., Delattre L., Brinker C.J., Lopez G.P. (1996). Amorphous silica molecular sieving membranes by sol-gel processing. Adv. Mater..

[13] De Vos R.M., Maier W.F., Verweij H. (1999). Hydrophobic silica membranes for gas separation. J. Membr. Sci..

[14] Iarikov D., Hacarlioglu P., Oyama S.T. (2011). Amorphous silica membranes for H_2_ separation prepared by chemical vapor deposition on hollow fiber supports. J. Membr. Sci..

[15] Gavalas G.R., Megiris C.E., Nam S.W. (1989). Deposition of H_2_-permselective SiO_2_ films. Chem. Eng. J..

[16] Ohta Y., Akamatsu K., Sugawara T., Nakao A., Miyoshi A., Nakao S.-I. (2008). Development of pore size-controlled silica membranes for gas separation by chemical vapor deposition. J. Membr. Sci..

[17] Nomura M., Aida H., Gopalakrishnan S., Sugawara T., Nakao S.-I., Yamazaki S., Inada T., Iwamoto Y. (2006). Steam stability of a silica membrane prepared by counter-diffusion chemical vapor deposition. Desalination.

[18] Miyajima K., Eda T., Naire B.N., Honda S., Iwamoto Y. (2013). Hydrothermal stability of hydrogen permselective amorphous silica membrane synthesized by counter-diffusion chemical vapor deposition method. J. Ceram. Soc. Jpn..

[19] Akamatsu K., Murakami T., Sugawara T., Kikuchi R., Nakao S.-I. (2011). Stable equilibrium shift of methane steam reforming in membrane reactors with hydrogen-selective silica membranes. AIChE J..

[20] Yoshino Y., Suzuki T., Nair B.N., Taguchi H., Itoh N. (2005). Development of tubular substrates, silica based membranes and membrane modules for hydrogen separation at high temperature. J. Membr. Sci..

[21] Akamatsu K., Nakane M., Sugawara T., Nakao S.-I. (2009). Performance under thermal and hydrothermal condition of amorphous silica membrane prepared by chemical vapor deposition. AIChE J..

[22] Smart S., Vente J.F., Diniz da Costa J.C. (2012). High temperature H_2_/CO_2_ separation using cobalt oxide silica membranes. Int. J. Hydrog. Energy.

[23] Karakiliç P., Huiskes C., Luiten-Olieman M.W.J., Nijmeijer A., Winnubst L. (2019). Sol-gel processed magnesium-doped silica membranes with improved H_2_/CO_2_ separation. J. Membr. Sci..

[24] Song H., Wei Y., Qi H. (2017). Tailoring pore structures to improve the permselectivity of organosilica membranes by tuning calcination parameters. J. Mater. Chem. A..

